# Multidimensional environmental influences on timing of breeding in a tree swallow population facing climate change

**DOI:** 10.1111/eva.12315

**Published:** 2015-10-23

**Authors:** Audrey Bourret, Marc Bélisle, Fanie Pelletier, Dany Garant

**Affiliations:** Département de biologie, Université de SherbrookeSherbrooke, QC, Canada

**Keywords:** climate change, density, laying date, phenology, phenotypic plasticity, temperature

## Abstract

Most phenological traits are extremely sensitive to current climate change, and advances in the timing of important life-history events have been observed in many species. In birds, phenotypic plasticity in response to temperature is thought to be the main mechanism underlying yearly adjustment in the timing of breeding. However, other factors could be important and interact to affect the levels of plastic responses between and/or within-individuals. Here, we use long-term individual-based data on tree swallow (*Tachycineta bicolor*) to identify the spatial and environmental drivers affecting plasticity in laying date and to assess their importance at both population and individual levels. We found that laying date has advanced by 4.2 days over 10 years, and that it was mainly influenced by latitude and an interaction between spring temperature and breeder density. Analyses of individual plasticity showed that increases in temperature, but not in breeder density, resulted in within-individual advances in laying date. Our results suggest that females can adjust their laying date as a function of temperature, but that this adjustment will be partly constrained in habitats with lower breeder densities. Such potential constraint is especially worrying for the broad array of species already declining as a result of climate change.

## Introduction

Effects of current climate change are ubiquitous and severely affect environmental conditions in wild populations (McCarty 2001; Parmesan and Yohe 2003; Walther 2010). Phenological traits are particularly sensitive to these environmental modifications, and as a result, over the last decades, phenological changes have been observed in several taxa from plants to mammals (Root et al. 2003; Menzel et al. 2006; Parmesan 2006; Thackeray et al. 2010; Poloczanska et al. 2013). However, the processes underlying observed phenotypic changes remain largely unknown, mainly because the distinction between mechanisms such as genetic changes and phenotypic plasticity is often unclear (Gienapp et al. 2008; Gienapp and Brommer 2014; Merilä and Hendry 2014). Consequently, our predictions of species adaptations to the ongoing environmental modifications remain elusive.

Phenotypic plasticity – the variation in the expression of phenotypes by a genotype in response to the environment (Bradshaw 1965; Stearns 1989) – is usually accepted as the main process to cope with environmental changes in the short term (Gienapp et al. 2008; Charmantier and Gienapp 2014; Gienapp and Brommer 2014; Merilä and Hendry 2014). However, studies have suggested that the importance and magnitude of phenotypic plasticity might be variable among populations (Husby et al. 2010; Porlier et al. 2012) and that the quality of its inference is relatively weak (Gienapp and Brommer 2014; Merilä and Hendry 2014). Importantly, multiple potential environmental drivers of the observed phenotypic changes are rarely studied exhaustively, despite the fact that more than one environmental factor may be affecting or constraining the plastic responses observed in wild populations (Merilä and Hendry 2014). Yet, by choosing *a priori* a single environmental driver, one can miss important causes of the observed phenotypic change (e.g. climate change versus habitat degradation) and predict inaccurate species response and/or suggest ineffective conservation actions to undertake (Charmantier and Gienapp 2014; Merilä and Hendry 2014). Finally, phenotypic plasticity can also be under selection and contribute to adaptive evolution, either directly through an underlying genetic basis or indirectly by allowing survival of populations in new environmental conditions and maintain them relatively close to new phenotypic optimum (Price et al. 2003; Brommer et al. 2005; Ghalambor et al. 2007; Nussey et al. 2007; Merilä and Hendry 2014). For all these reasons, investigating the importance of phenotypic plasticity, in terms of assessing individual and population variations, its environmental drivers and its influence in observed phenotypic trends, is a critical first step to obtain a more complete understanding of evolutionary processes underlying phenotypic changes caused by current climate change.

Different environmental and spatial drivers can affect plasticity of phenological traits, either directly by acting as cues of future environmental conditions or indirectly through population differentiation captured in space and/or by acting as constraints on plastic responses. Physiological regulation of phenological events in birds comes from the integration of diverse cues from which photoperiod is the most important because its perception allows an annual read of time passing (Sharp 2005; Bradshaw and Holzapfel 2007; Dawson 2008; Visser et al. 2010). Annual photoperiod variation increases with latitude and could explain most of within-species latitudinal variation in life-history events (Lambrechts et al. 1997; Bradshaw and Holzapfel 2007; Dawson 2013). Finer adjustments (i.e. plasticity) are allowed by the integration of other environmental signals from the physical and social environments (Ball and Ketterson 2008; Dawson 2008). For instance, temperature is thought to be the main driver of timing of breeding in birds (Meijer et al. 1999; Visser et al. 2009; reviewed in Caro et al. 2013), but other factors such as rainfall, often a cue for food availability (Hau 2001; Saunders et al. 2013), and social interactions (Caro et al. 2007) have been reported to play a role in some populations. Knowledge of how these various cues are perceived by the circadian system is still scarce (Dawson 2008), as is appreciation of variation in the perception of these multidimensional cues among individuals (i.e. IxE) or populations (Lyon et al. 2008; Visser 2008; Visser et al. 2010). These cues may also interact with other environmental components and constrain the levels of plastic responses displayed between and/or within-individuals (Wilson et al. 2007). However, very few studies have addressed these possible interactive effects.

Here, we use 10 years of data from a tree swallow (*Tachycineta bicolor*) long-term study to investigate the role of multiple spatial (latitude, longitude and elevation) and environmental (spring temperature, rainfall and breeder density) determinants of laying date. We first assess the influence of potential factors and their interactions on laying date at the population level in our 10 200-km^2^ study system. These factors were chosen based on previous knowledge of their potential influence on laying date in tree swallows and other bird species. We then examine the importance of these factors at both population (among-individuals) and individual (within-individuals) levels of plasticity. The tree swallow is a small migratory passerine, an aerial insectivorous, and it produces only one clutch per year, all characteristics of species more at risk under current climate changes (Both and Visser 2001; Møller et al. 2008; Dunn and Winkler 2010; Thackeray et al. 2010; Dunn and Møller 2014). In fact, tree swallow populations are severely declining in the eastern part of their distribution (Nebel et al. 2010; Shutler et al. 2012), including in our study area (Rioux Paquette et al. 2014). However, the causes for these declines are still unknown despite some indications pointing at agricultural intensification in breeding areas (e.g. Ghilain and Bélisle 2008; Rioux Paquette et al. 2013) or at carry-over effects from nonbreeding areas (e.g. Rioux Paquette et al. 2014; but see also Dunn et al. 2011 and Dunn and Møller 2014).

The mean laying date of tree swallows has also advanced in most populations across the continent over the last five decades (Dunn and Winkler 1999, 2010; Rioux Paquette et al. 2014; but see Hussell 2003 for an exception). A previous analysis in our study system showed that selection favoured earlier laying date in this population but that patterns of selection fluctuated in strength and direction through time (Millet et al. 2015). Also, the time lag observed in the studied area between spring arrival (eBird, http://ebird.org/) and reproduction suggests that further adjustments of laying date are possible. Latitude, spring temperature and breeder density (as a proxy of habitat quality) were suggested to influence tree swallow laying date at a large spatial scale (Dunn and Winkler 1999; Winkler et al. 2002), but we have little knowledge of other potential environmental and spatial factors, their influences at a small spatial scale and their relative importance on population and individual levels of plasticity.

## Methods

### Study system and data collection

**Figure 1:**
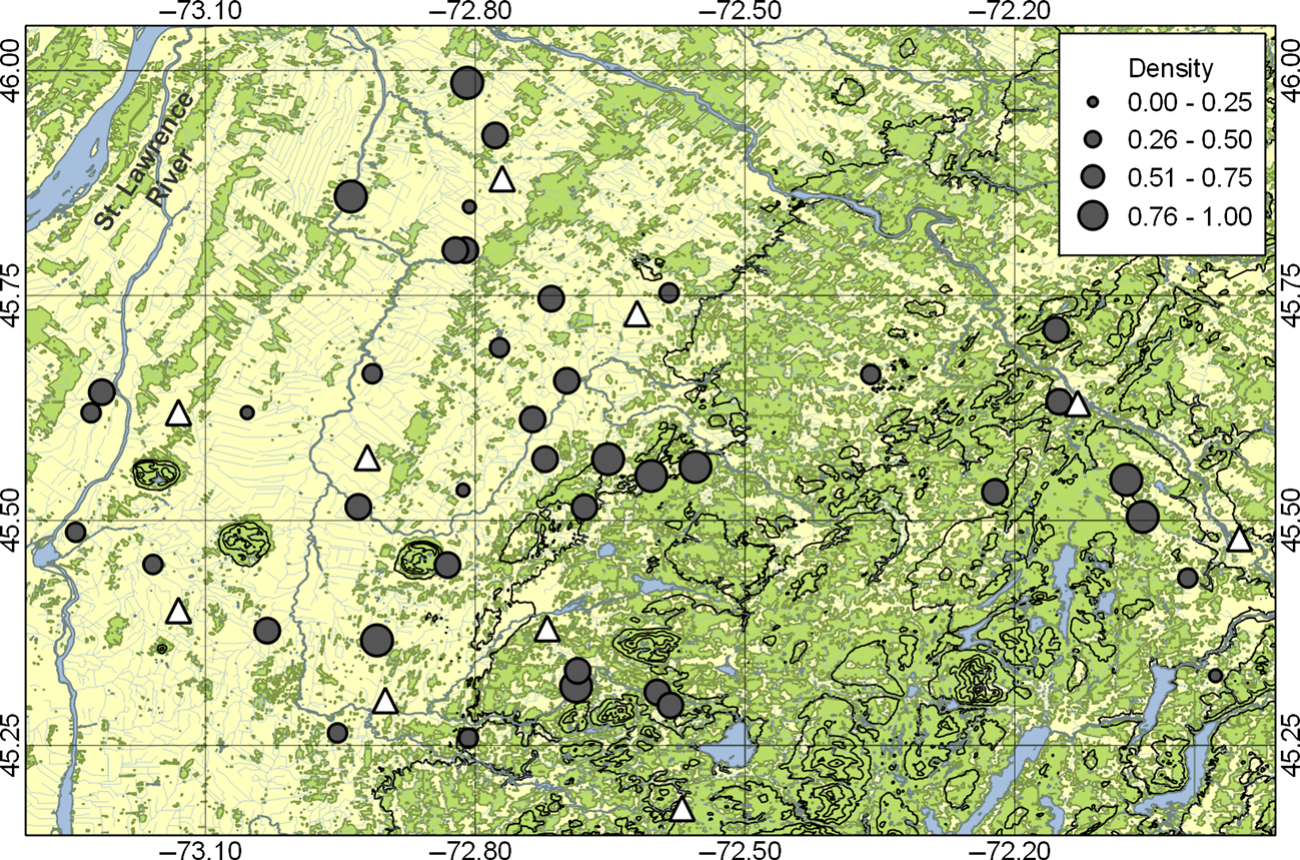
Distribution of the 40 farms (grey circles) and 10 meteorological stations (white triangles) in the study system in southern Québec. Mean density of breeders on a farm (% of occupied nest boxes) between 2004 and 2013 is represented by different circle sizes (see legend). Forest patches (green), rivers and lakes (blue), other land uses (mostly agriculture; yellow), elevation (100-m black isolines), latitude and longitude (in decimal degrees; thin black lines) are also represented. This figure was created with QGIS 2.0 (QGIS Team Development 2013).

Between 2004 and 2013, during the breeding season (April to August), we monitored 400 nest boxes within 40 farms (10 nest boxes per farm, separated by 50 m, thus covering similar areas on each site) in southern Québec, Canada (covering an area of 10 200 km²) (Fig. 1; see Ghilain and Bélisle 2008 for more details on the study system). During this period, each nest box was visited every 2 days to record occupancy and laying date of the first egg (in Julian days; January 1 = Julian day 1). Females were captured during the incubation period, while males were caught during the nestlings’ food provisioning phase. All tree swallows were individually identified with an aluminium band (US Fish and Wildlife Service). Females were aged based on feather colour: brown females were assigned to second-year class (SY) and blue-green females to after second-year class (ASY) (Hussell 1983). Since 2006, the sex of every individual was confirmed with a molecular technique following Lessard et al. (2014). In our analysis, we only considered first clutches, that is first breeding event in a nest box of both female and male (if known) within a reproductive season (*n* = 2273; see Table A1 for details on yearly sample sizes). Second clutches are rare (12.7% of all clutches) and mostly result from first clutch failures.

Spring temperature (°C) and rainfall (mm) data were obtained in two steps, using information collected from meteorological stations located within the study area (obtained from Environment Canada, http://meteo.gc.ca/; Table A2; Fig. 1). First, a sliding windows approach was used to determine the most relevant time period suitable for all farms for these two meteorological variables and to guard against potentially misguided *a priori* choices (see Brommer et al. 2008 and Porlier et al. 2012 for similar approaches). For this analysis, we used a unique climatic variable value obtained by averaging values from the three meteorological stations nearest from the centroid of our study system (centroid: 45.57°N, −72.64°W; Table A2). We tested windows varying from 5 to 91 days, from Julian days 60 to 151 (respectively, March 1 and May 31 in nonleap year) for a total of 3828 windows. Pearson’s correlations between annual mean of averaged daily value for each window and annual mean laying dates were used to determine the most relevant period for each environmental variable. The strongest correlation between mean temperature and mean laying date was found between Julian day 96 and 129 (April 6–May 9; *r *=* *−0.750, *P *=* *0.012), while for rainfall, this window was between Julian day 128 and 133 (May 8–13; *r *=* *−0.748, *P *=* *0.013). As a second step, we used these periods as our references for computing both annual mean temperatures and annual rainfalls (hereafter spring temperature and rainfall) from 10 meteorological stations near our farms (Fig. 1; Table A2; distances range between each farm and the nearest meteorological stations: 1.6–20.1 km), allowing at the same time a fine resolution of the spatial and temporal environmental variation across the study system and a comparison of laying dates among farms in the plasticity analyses.

### Environmental determinants of laying date at the population level

We used the annual mean laying dates for each farm (*n* = 392 as no birds were observed in 8 farm-years; *r *=* *0.92 between annual mean and median laying dates) to assess both the temporal (interannual) trend in laying and the environmental determinants of laying date. For the temporal trend, we used a linear mixed model to estimate the annual change in mean laying date over the study period (10 years), with farm identity included as random effect. Then, we fitted a linear mixed model to quantify the effects of different environmental variables on mean laying date. The full model included spring temperature, rainfall, breeder density (% of the 10 nest boxes on each farm occupied), elevation (m) and latitude (decimal degree) and all two-way interactions as fixed effects (see also Table A3 for the range limit of each environmental component). We did not include longitude and distance from the St. Lawrence River as they were both highly correlated with elevation (*r *>* *0.9; Fig. 1) (see also Porlier et al. 2009). All explanatory variables were standardized (zero mean, unit variance; Table A3) to facilitate the interpretation of their relative influence on mean laying dates. Year and farm identity were tested as random effects using likelihood ratio tests (LRTs), but only year was significant and kept in analyses (but see Table A4 for a model including both year and farm identity as random effects – the selected final model and its effect sizes were similar in both cases).

### Individual plasticity in laying date

Individual plasticity in laying date was modelled including only two of three environmental variables that were significant in the population-level analysis (i.e. spring temperature, breeder density; see Results). Although latitude was significant at the population level (see Results), it was not an appropriate variable to assess individual plasticity because it has limited variation for a given individual over its lifetime. In fact, tree swallows can be considered philopatric to their breeding site in our study area as only 8.1% of our observations were indicative of females having dispersed between farms (*n* = 1015 observations on 397 females, among different breeding events; see Lagrange et al. 2014). All environmental variables were standardized (zero mean, unit variance; Table A3). Age was included as a covariate in our models because of its influence on laying date: older females reproduce earlier than younger ones (Stutchbury and Robertson 1988; Bentz and Siefferman 2013; this study, see Results), and thus, females sampled in 2004 were excluded as we had no information about their age.

We first assessed the relationship between the difference in laying dates (laying date year 2 – laying date year 1) and the difference in environmental conditions between years (environmental value year 2 – environmental value year 1) for all females breeding in two consecutive years. This analysis was conducted using a linear model and was repeated for three data sets: (i) females observed as SY on the first year (*n* = 63, refer to as the SY dataset), (ii) females observed as ASY on both years (*n* = 311, refer to as the ASY dataset) and (iii) all females with age class on the first year as fixed effect (*n* = 349, refer to as the total dataset). For females breeding in more than two years, we included only the first two consecutive observations in these analyses.

We then investigated individual plasticity and between-individual variation in plasticity (IxE) with a random regression analysis (Nussey et al. 2007) on females that were observed in at least two years between 2005 and 2013 (*n* = 935 observations on 370 females). We compared increasing structure complexity of random effects (year, farm, female identity) with LRTs, including random slopes with environmental variables (IxE). Furthermore, because not all individuals experienced the same set of environmental conditions, we used the within-subject centring technique for environmental variables to separate individual variation from population trend (Kreft et al. 1995; Snijders and Bosker 1999; van de Pol and Wright 2009). Hence, each environmental variable (temperature and breeder density) was subdivided into a within-individual (*β*_W_) and a between-individual (*β*_B_) component. Briefly, for each female, we calculated a mean value of temperature and breeder density experienced (i.e. between-individual effect, reflecting the population trend), and for all observations, an individual deviation from these mean values (i.e. within-individual effect, reflecting individual plasticity). The full model included as fixed effects within-individual (*β*_W_) and between-individual (*β*_B_) components of both spring temperature and breeder density and also female age class and latitude to control for their effects. Best linear unbiased predictors (BLUPs) for each female (i.e. individual slope and elevation) were generated from the final model to graphically represent individual-specific plastic response.

All statistical analyses were conducted in the R statistical environment 3.0.2 (R Core Team 2014). Linear mixed model analyses were performed using the lme4 package (Bates et al. 2014). Degrees of freedom (Satterhwaite’s approximation) and *P*-values of mixed models were calculated using the lmerTest package (Kuznetsova et al. 2013). Final models were determined by sequentially removing the least significant term from the model based on its *P*-value and comparing with a LRT this new model to the previous one, repeatedly until all remaining variables were significant (*α *= 0.05) (Crawley 2007).

## Results

### Phenological changes and environmental determinants

**Figure 2:**
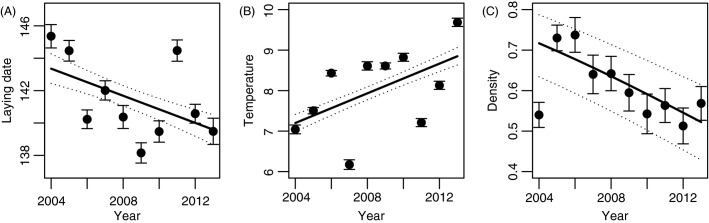
Temporal trend at the population level in (A) mean laying date (Julian days) of tree swallows, (B) spring temperature (°C) and (C) density of breeders (% of occupied nest boxes) over the 40 farms monitored between 2004 and 2013. Black circles depict mean values (±SE) over all farms for each year, and black lines are model predictions (dotted lines: 95% CI).

**Figure 3:**
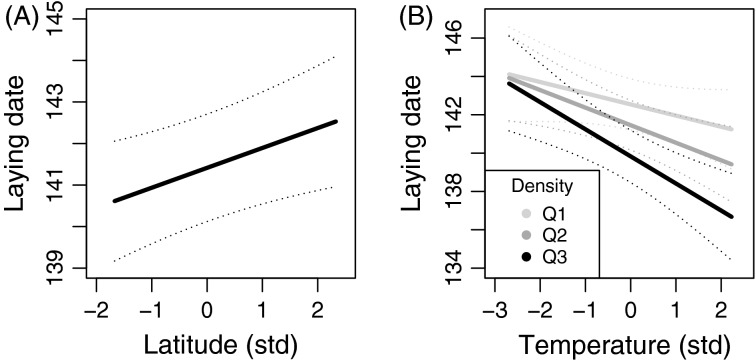
Predictions from the linear mixed model of environmental determinants of tree swallow laying date at the population level for A) latitude and B) the interaction between spring temperature and breeder density [first (Q1, lowest), second (Q2) and third (Q3, highest) quartile of density values presented]. See Table1 for details.

Tree swallow annual mean laying date advanced by approximately 4.2 days over the 10-year study period (*β* = −0.419 ± 0.076, *t* = 5.50, *P *<* *0.001; Fig. 2A). Further analyses revealed an increase in spring temperature (*β* = 0.183 ± 0.017, *t* = 11.09, *P *<* *0.001; Fig. 2B) and a decrease in breeder density (*β* = −0.093 ± 0.014, z = 6.83, *P *<* *0.001; Fig. 2C) over the same period (linear mixed model and generalized linear mixed model (logit link and binomial error) were used, respectively, with farm identity included as a random effect). The final model of the environmental determinants of laying date included latitude and an interaction between mean temperature and breeder density as significant explanatory variables (Table1). More specifically, farms at higher latitudes (northern locations) showed later mean laying dates than those at lower latitudes (Table1; Fig. 3A). Laying date was also earlier when spring temperature increased; this relationship was steeper under higher breeder density (Table1; Fig. 3B). Rainfall and elevation did not significantly affect laying date and thus were not kept in the final model.

**Table 1:** Final linear mixed model at the population level of the environmental determinants of mean laying date in tree swallows (*n* = 392). Environmental variables have been standardized prior to the analysis. Year was included as random effect. Adjusted *R*^2^ for fixed effects was 0.182

Variable	Estimate	SE	d.f.	*t*-value	*P*-value
Intercept	141.415	0.658	8.1	215.01	<0.001
Latitude	0.479	0.195	376.9	2.46	0.014
Breeder density	−1.469	0.205	383.4	7.16	<0.001
Temperature	−0.929	0.341	158.5	2.73	0.007
Temperature × Breeder density	−0.450	0.204	379.6	2.20	0.028

### Individual plasticity in laying date

Our analyses showed evidence of individual plasticity as a function of spring temperature but not of breeder density. The first analysis of individual plasticity showed negative slopes for change in laying date as a function of temperature differential for all three datasets (i.e. SY, ASY and total dataset; Table2). This result suggested that an increase in temperature between years resulted in earlier laying date over the same period. Contrastingly, analyses of change in laying date as a function of differences in breeder density revealed nonsignificant negative trends with earlier laying dates at higher densities for the all data sets (Table2). Finally, SY females laid their eggs more than five days later than ASY ones (Table2).

**Table 2:** Individual-based analyses of plasticity quantifying the change in laying date between two consecutive years by female tree swallows in relationship to change in spring temperature and breeder density for (a) females observed as SY on the first year (*n* = 63), (b) females observed as ASY in both years (*n* = 311), (c) all females (*n* = 349; age was included as fixed effect)

Model	Variable	Estimate	SE	*t*-value	*P*-value
a) SY	**Intercept**	**−8.159**	**1.246**	**6.55**	**<0.001**
*R*² = 0.113	**ΔTemperature**	**−3.742**	**1.256**	**2.98**	**0.004**
	ΔDensity	−1.111	1.262	0.88	0.38
b) ASY	**Intercept**	**−2.415**	**0.393**	**6.14**	**<0.001**
*R*² = 0.099	**ΔTemperature**	**−2.338**	**0.394**	**5.94**	**<0.001**
	ΔDensity	−0.629	0.394	1.60	0.11
c) TOTAL	**Intercept**	**−2.349**	**0.448**	**5.24**	**<0.001**
*R*² = 0.158	**Age**	**−5.683**	**1.056**	**5.38**	**<0.001**
	**ΔTemperature**	**−2.462**	**0.407**	**6.06**	**<0.001**
	ΔDensity	−0.764	0.406	1.88	0.061

Variables in bold characters were kept in final models, and adjusted *R*² values are presented.

**Figure 4:**
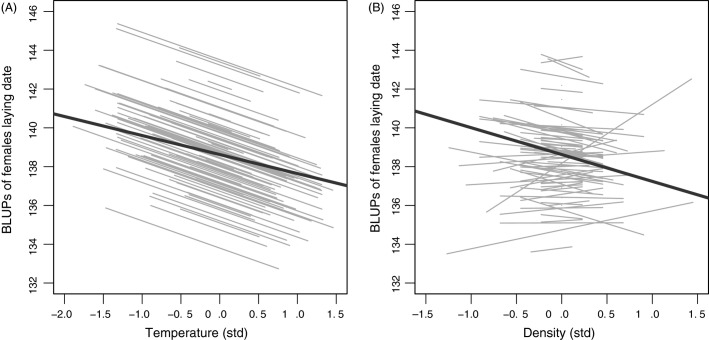
Best linear unbiased predictions (BLUPs; grey lines) for 100 female tree swallows (randomly chosen over a possibility of 370) from the random regression model (model 5, Table3) of individual plasticity in laying date (Julian days), for within-individual component (*β*_W_) of standardized (A) spring temperature and (B) breeder density. Bold black lines represent predictions from between-individual components (*β*_B_).

The random regression analysis first showed evidence for individual slopes variability in the relationship between laying date and breeder density in the random part of the model (i.e. IxE for breeder density; model 5: LRT = 10.81, *P *=* *0.004; Table3; Fig. 4A), but not for individual-by-temperature variability (i.e. no IxE for spring temperature; model 4: LRT = 0.50, *P *=* *0.78; Table3; Fig. 4B). Estimates of the within-individual (*β*_W_) and between-individual (*β*_B_) components of environmental variables showed different pattern for spring temperature and breeder density effects (Table3; Fig. 4). For spring temperature, both *β*_W_ and *β*_B_ showed a significant negative relationship – with earlier laying date at warmer temperature. However, for breeder density only the between-individual component was significant and negative, suggesting that the earlier laying dates at higher breeder density reflected a difference at the population level but no individual plasticity. Finally, the comparison between estimates of within-individual and between-individual slopes within each environmental variable suggested no significant difference between temperature components (*β*_W_ = *β*_B_, *P *=* *0.38) and a significant difference between breeder density components (*β*_W_ ≠ *β*_B_, *P *=* *0.039) (Table A5; see equation 2 in van de Pol and Wright 2009 for more details on the technique used).

**Table 3:** Random regression analyses of the effect within-individual (*β*_W_) and between-individual (*β*_B_) components of two environmental variables, spring temperature and density of breeders, on female tree swallow laying dates (*n* = 935 observations on 370 females). Random structures of model 1 to 5 were compared with a LRT. Estimates of fixed effects and variance components of random effects of model 5 (random slope function of breeder density) are presented. Within-individual centring technique (*β*_W_ vs *β*_B_) was applied as suggested by van de Pol and Wright (2009)

Models	Log-L	Test	d.f.	LRT	*P*-value
1. Year	−2911.0		9		
2. Year + Farm	−2903.9	1 vs 2	10	14.13	<0.001
3. Year + Farm + Female	−2885.9	2 vs 3	11	36.12	<0.001
4. Year + Farm + Female × Temperature_within_	−2885.6	3 vs 4	13	0.50	0.78
5. Year + Farm + Female × Density_within_	−2880.4	3 vs 5	13	10.81	0.004

Fixed effects	Estimate	SE	d.f.	*t*-value	*P*-value	Random effects	Var	Corr
Intercept (*β*_0_)	138.605	0.752	7.7	184.41	<0.001	Female (intercept)	7.660	
Age	7.274	0.646	838.7	11.25	<0.001	Density_within_ (slope)	7.488	−0.20
Latitude	0.638	0.303	26.9	2.11	0.045	Year (intercept)	4.243	
Temperature_within_ (*β*_W_)	−1.408	0.468	76.5	3.01	0.004	Farm (intercept)	1.563	
Temperature_between_ (*β*_B_)	−0.995	0.470	88.4	2.12	0.037	Residual	19.092	
Density_within_ (*β*_W_)	−0.347	0.421	175.0	0.82	0.41			
Density_between_ (*β*_B_)	−1.386	0.297	105.0	4.67	<0.001			

The observed population trend (i.e. *β*_B_) as function of breeder density – without a significant within-individual component – and the observation of steeper laying date–spring temperature slope with increasing breeder density in the environmental determinant analysis suggested that females living on average at lower densities were possibly constrained in their plastic response. To further explore the hypothesis that lower density farms imposed a constraint on laying date plasticity (in response to spring temperature), we conducted additional individual plasticity analyses using data sets subdivided into high and low breeder densities (see Appendix S2). We found that for both individual plasticity analyses (i.e. change in laying date and random regression analysis) plastic responses to temperature were slightly more negative in the high density than in the low density subset (Table B1–B3), which could potentially be explain a stronger plastic response at higher density of breeders.

## Discussion

In this study, we were interested in the multidimensional influence that environmental variation can have on phenological traits, even at a small spatial scale. Here, we have shown the importance of three environmental variables – latitude, spring temperature and breeder density – and found evidence of individual plasticity as a function of spring temperature but not of breeder density and no evidence of variation in individual slopes. Our results also suggested that females breeding on average in areas of lower individual densities were possibly constrained in their adjustment of laying date in response to spring temperature.

### Phenological change

Tree swallows in our population have advanced their annual mean laying date by about 0.42 day/year over the 10-year study period. This rate of advance is higher than the 0.28 day/year advance that was previously reported for this species throughout North America (study period: 1959 to 1991, Dunn and Winkler 1999). This difference can be explained by either an increase in this rate in the last two decades or by geographic variation in effects and/or responses to climate change (e.g. Hussell 2003; Dunn and Møller 2014). However, these two potential explanations could only be distinguished by performing a new temporal trend analysis of tree swallow laying dates across their range. The observed advancement is also greater than the mean trend computed from several long-term studies on birds (mean advance of 0.13 day/year, *n* = 68 species, Dunn and Winkler 2010), but is still comparable to observations from a few previous studies on migrant species (e.g. eurasian reed warblers (*Acrocephalus scirpaceus*): advance of 0.48 day/year, Crick and Sparks 1999; great reed warblers (*Acrocephalus arundinaceus*): advance of 0.55 day/year, Dyrcz and Halupka 2009).

### Environmental determinants

Numerous previous studies in birds reported within-species latitudinal variation in phenology, reflecting different readings of photoperiod (e.g. Sanz 1998; Dunn and Winkler 1999; Gienapp et al. 2010; reviewed in Dawson 2013). However, the latitudinal variation in laying date documented here is particularly striking given the small spatial scale involved (80-km span in latitude) compared to previous studies (e.g. North American continent, Dunn and Winkler 1999; 700-km span in latitude, Gienapp et al. 2010). Our result may be partly explained by larger day length variation in space than in time during the breeding season in this region. For instance, on May 20th (the mean laying date across all observations in our study; Julian day 140 in nonleap years), the difference in day length between the most distant sites in terms of latitude in our study system was of approximately 5 min, while the difference between two consecutive days was around 2 min (calculated with the NOAA solar calculator, http://www.esrl.noaa.gouv). Considering that 30–60 min changes in day length over an entire year can be perceived as cues for breeding and moulting in bird species distributed near the Equator (Hau 2001; Goymann et al. 2012), it is plausible that the latitude effect on laying date documented here partly reflects a difference in day length captured by the circadian rhythm of individuals.

Variation in density of breeders is rarely studied as a potential determinant of timing of breeding in birds, but it showed the largest effect size on mean laying date. The negative relationship we observed – later laying date at lower density – is similar to observations from other tree swallow populations (models using species abundance indices from the Breeding Bird Survey programme, Dunn and Winkler 1999; Winkler et al. 2002), but contrary to expectations under intraspecific resource competition (e.g. Wilkin et al. 2006; Wilson et al. 2007; but see also Ahola et al. 2012 for a special case where intraspecific resource competition lead to earlier laying date). Dunn and Winkler (1999) suggested that differences in habitat quality should lead to an aggregation of individuals in areas with more food, while areas with fewer resources should limit and constrain laying date (e.g. food availability, Shorrocks et al. 1998; Robb et al. 2008). This is supported by the positive correlation usually observed between nest box occupancy rate and insect abundance (Hussell 2012), and by the negative correlation observed between timing of breeding and flying insect biomass during the laying period (Dunn et al. 2011) in tree swallows. Tree swallows do not follow an ideal-free distribution in our study area as birds nesting in low-quality habitats have smaller clutch sizes and lower reproductive success (Ghilain and Bélisle 2008; Lessard et al. 2014). We could also speculate that the observed relationship is partly explained by the activity of the circadian system, where the density of breeders could act, similar to the effect of temperature, as an environmental cue (e.g. the presence of conspecific may be needed to initiate breeding events as in Caro et al. 2007) regulating timing of breeding in females (Dawson 2008). Nevertheless, our detailed analyses of individual plasticity do not support this last hypothesis.

Temperature is usually proposed to be the most important environmental variable determining laying date in birds (Visser et al. 2009; Caro et al. 2013). In our sliding window analysis, the temperature during the month preceding the laying period was providing the strongest correlation. This period is similar to what has been observed in other bird species (e.g. common gulls (*Larus canus*), Brommer et al. 2008; great tits (*Parus major*), Husby et al. 2010; blue tits, Porlier et al. 2012) and corresponds to the period of increasing spring temperatures acting directly as a signal for the timing of breeding in birds (Visser et al. 2009; Schaper et al. 2012). Indeed, a tendency for earlier timing of breeding at higher spring temperature has been observed in several bird species (Dunn and Winkler 2010; Charmantier and Gienapp 2014), including tree swallows (Dunn and Winkler 1999; Winkler et al. 2002; this study). The temperature–density interaction observed, with more negative laying date–spring temperature slope at higher breeder density, further supports the environment quality hypothesis, as at lower densities of breeders (lower quality habitats) it might be harder for individuals to respond to environmental cues and effectively adjust their laying date (see also discussion on individual plasticity below).

### Individual plasticity and between-individual effect

Evidence of individual plasticity in laying date in response to spring temperature in both plasticity analyses suggests that this environmental variable may potentially act as a cue for timing of breeding in tree swallows. Our first observation that changes in temperature experienced by a female will lead to changes in its timing of breeding has been supported by the within-subject centring technique where individual plasticity (within-individual component, *β*_W_) remained significant despite the heterogeneity observed in sampling (between-individual component, *β*_B_). It is possible that different mechanisms drive the patterns observed at the population and individual levels even if the trends are similar in direction and magnitude. However, the similarity in coefficients for within- and between-individual spring temperature components potentially suggests that the population trend observed can be explained by individual phenotypic plasticity (see Brouwer et al. 2013 and Gienapp and Brommer 2014 for similar interpretations when *β*_W_ = *β*_B_).

Density of breeders in our study system is probably not a social cue for reproductive timing, but could instead reflect a variation in individual capacity to initiate breeding linked with habitat quality. Our first individual plasticity analysis has shown no effect of variation in breeder density on individual laying date adjustment, and this finding was further supported by our second analysis showing that the within-individual component (*β*_W_) was not different from zero (i.e. no individual plasticity). These results combined with the observed negative population trend (*β*_B_) in laying date in our data suggested that changes in density a female will experience across breeding seasons will not affect her plastic response (i.e. not act as an environmental cue for timing of breeding) and that all females living on average at higher densities laid their eggs earlier (and *vice versa*). The possible constraint on plasticity for females at lower densities (lower quality habitats) suggested from the steeper laying date–spring temperature slope with increasing breeder density in the population-level analysis was further supported by the slightly stronger individual plastic response of laying date as function of temperature observed at high densities in our complementary analyses (Table B1–B3). Environmental constraints on phenotypic plasticity have also been described in song sparrows (*Melospiza melodia*) on Mandarte Island (British Columbia, Canada), where cohorts born in better environmental conditions showed higher plastic response in response to the El Niño Southern Oscillation (Wilson et al. 2007). While we believe that the pattern described here is likely to be nonadaptive, given that tree swallows breeding later show a reduced fitness in most years (Millet et al. 2015), further investigations are needed to clearly conclude on the effects of reduced plasticity in lower density habitats (e.g. compare selection gradients between low and high breeder density farms).

Variability in individual responses to the environment (IxE) is considered the raw material for phenotypic plasticity evolution (Nussey et al. 2007). In birds, IxE for laying date in response to temperature has been observed in most populations studied (reviewed in Gienapp and Brommer 2014). Here, the absence of IxE for spring temperature (i.e. no phenotypic variation in slopes), along with similar plastic responses at the population and individual levels, suggests that tree swallows can track temperature changes, probably as long as the observed variation is within the usual range of temperatures they are adapted to. The presence of IxE is usually tested by stepwise model building, where improvement in model likelihood when adding the IxE component is sufficient to suggest variation in the slope and thus individual variation in plasticity. Our results questioned this approach of assessing IxE. A first problem with this approach is the fact that an improvement to the model could be mainly due to the presence of a significant covariance between the slope and intercept rather than to a significant IxE interaction. Also, while we observed no variation in the slope for the spring temperature reaction norm, we observed individual variation in slope for breeder density (model 5, Table3), but no direction or pattern in the way individuals respond to variation in breeder density (i.e. *β*_W_, individual plasticity). Previous studies argued that heterogeneity in residual variance could lead to an over-estimation of IxE (Brommer 2013; Nicolaus et al. 2013), a phenomenon that cannot be discarded here. For all these reasons, the presence of a significant IxE interaction involving breeder density as random individual responses may not be representative of variability in phenotypic plasticity at the individual level.

### Applications of our study

Phenotypic plasticity in response to spring temperature can be an effective way for birds to keep adequate timing of life-history events in the face of climate change (reviewed in Charmantier and Gienapp 2014). For example, Vedder et al. (2013) have shown with a population persistence model that the actual level of individual plasticity in timing of breeding observed in great tits of Wytham Woods (UK) lowers their extinction risk by about 500-fold. However, the success of a population response to climate change via phenotypic plasticity can depend on many other environmental components. For example, degradation of environmental conditions in a Finnish population of pied flycatchers (*Fiducela hypoleuca*) is suspected to be a cause for the observed mismatch between breeding time and phenology of the environment (Laaksonen et al. 2006). Studying all potential factors influencing phenological traits is crucial for a more complete understanding of the potential of phenotypic plasticity to adequately track environmental changes. Here, our initial choice of environmental variables was based on factors previously shown to influence tree swallow laying date, but was also guided by data availability. Ideally, we should have used a measurement of habitat quality (e.g. food availability) rather than a proxy (i.e. breeder density) and also a finer measurement of climatic variables (e.g. temperature and rainfall for each farm). Yet, using the best proxy available is arguably a better option than not taking it into account when analysing plasticity.

Environmental conditions have changed over the study period in our system, with both an increase in spring temperature and a diminution in breeder density (see also Rioux Paquette et al. 2014). These changes influenced the phenological response to environmental cues in contrasting ways. While we found a phenotypically plastic response for changes in spring temperature, the more limited capacity to respond to temperature cues (i.e. reduced individual plasticity) that we suspect in lower density habitats is worrying for tree swallow populations in the context of concurrent climate change, population decline and reduced fitness for individuals breeding later (Millet et al. 2015). Multiple environmental drivers of phenotypic changes can act in synergy and accelerate the rate of extinction (Brook et al. 2008). Unfortunately, models predicting species response to climate change rarely included phenotypic plasticity, population-level response and/or multidimensional environmental factors despite evidences of important bias caused by such omissions (Chevin et al. 2010; Reed et al. 2011; Bellard et al. 2012; Valladares et al. 2014). If plastic responses are constrained in lower quality habitats, and that several human-driven changes are occurring simultaneously, the ability of species to respond to climate change may be jeopardized and lead to further biodiversity loss. Studies such as this one are still necessary to improve our knowledge of the effects of important environmental factors, to understand how they interact together and to assess, rather than assume, the importance of plastic responses underlying observed phenotypic changes. Altogether, our results enlighten the complexity of phenotypic plasticity as a way for populations to cope with current climate change.

## Data archiving statement

Data available from the Dryad Digital Repository: http://dx.doi.org/10.5061/dryad.87jb3.

## Supporting Information

**Appendix S1.** Supplementary information on methods and results. **Table A1.** Sample sizes of clutches and female tree swallows (for first breeding attempt only) between 2004 and 2013. **Table A2**. Information on the meteorological stations used in the analysis. **Table A3**. Descriptive statistics of environmental variables included in the statistical analyses prior to standardization, for (A) the environmental determinants analysis (see table 1), (B) the change in laying date analysis (see table 2) and (C) the random regression model (see table 3). **Table A4**. Linear mixed effects model used to assess if slopes of within-individual (β_W_) and between-individual environmental components (β_B_) are similar or not (following van de Pol and Wright [1], equation 3; see also table 3). **Appendix S2.** Additional individual plasticity analyses. **Table B1**. Analyses of change in laying date between two consecutive years by female tree swallows in relationship to change in spring temperature and breeder density for a subset of (A) low density of breeders (158 females) and (B) high density of breeders (173 females). **Table B2**. Analyses of change in laying date between two consecutive years by female tree swallows in relationship to change in spring temperature and breeder density for a subset of (A) low density of breeders (87 females) and (B) high density of breeders (80 females). **Table B3**. Random regression analyses of the effect within-individual (β_W_) and between-individual (β_B_) components of spring temperature on female tree swallow laying dates for a subset of (A) low density of breeders (434 observations on 176 females) and (B) high density of breeders (456 observations on 175 females).
