# Supplementary material for: Multidimensional environmental influences on timing of breeding in a tree swallow population facing climate change
Source: Evol Appl. 2015 Oct 23;8(10):933–44. doi: 10.1111/eva.12315 (PMC4662344; doi:10.1111/eva.12315)
Supplement: Supplementary file 1 [file eva0008-0933-sd1.doc]

**SUPPORTING INFORMATION**

**Multidimensional environmental influences on timing of breeding in a tree swallow population facing climate change**

Audrey Bourret, Marc Bélisle, Fanie Pelletier and Dany Garant

**Appendix S1:** *Supplementary information on methods and results*

**Table A1** – Sample sizes of clutches and female tree swallows (for first breeding attempt only) between 2004 and 2013.

|  | **2004** | **2005** | **2006** | **2007** | **2008** | **2009** | **2010** | **2011** | **2012** | **2013** | **Total** |
| --- | --- | --- | --- | --- | --- | --- | --- | --- | --- | --- | --- |
| Clutches | 216 | 292 | 295 | 256 | 244 | 226 | 217 | 214 | 200 | 216 | 2376 |
| Females | 180 | 257 | 250 | 217 | 212 | 186 | 198 | 181 | 180 | 172 | 2033 |
| SY | - | 46 | 17 | 19 | 30 | 28 | 35 | 36 | 40 | 34 | 285 |
| ASY | - | 211 | 233 | 198 | 182 | 158 | 163 | 145 | 140 | 138 | 1568 |

**Table A2** – Information on the meteorological stations used in the analysis. Station name and ID refer to Environment Canada unique identification (<http://meteo.qc.ca/>), and underlined stations were used in the sliding windows analysis. Latitude and longitude are in decimal degrees, and the number of farms closest to each meteorological station is also reported.

| **Station name** | **Station ID** | **Latitude** | **Longitude** | **N closest farms** |
| --- | --- | --- | --- | --- |
| Brome | 7020840 | 45.18 | -72.57 | 2 |
| Bromptonville | 7020860 | 45.48 | -71.95 | 3 |
| Farnham | 7022320 | 45.30 | -72.90 | 3 |
| Granby | 7022800 | 45.38 | -72.72 | 6 |
| Marieville | 7024627 | 45.40 | -73.13 | 3 |
| Richmond | 7026464 | 45.63 | -72.13 | 5 |
| St-Guillaume | 7027302 | 45.88 | -72.77 | 5 |
| Ste-Madeleine | 7027517 | 45.62 | -73.13 | 3 |
| Ste-Hyacinthe | 7027361 | 45.57 | -72.92 | 3 |
| St-Nazaire | 7027588 | 45.73 | -72.62 | 7 |

**Details on treatment of meteorological data**: From the raw data of Environment Canada, daily value with indicator “E” (i.e., estimated) and “C” (i.e., precipitation occurred, amount uncertain) were excluded. When more than five consecutive daily values were missing, they were replaced with those from the closest meteorological station (7 replacement periods over 10 years of data from 5 meteorological stations).

**Table A3** – Descriptive statistics of environmental variables included in the statistical analyses prior to standardization, for A) the environmental determinants analysis (see table 1), B) the change in laying date analysis (see table 2) and C) the random regression model (see table 3).

| **Analysis** | **Environmental variable** | **Range** | **Mean** | **Standard deviation** |
| --- | --- | --- | --- | --- |
| A) Environmental determinants | Density | 0.1 – 1.0 | 0.6 | 0.3 |
|  | Latitude | 45.26 – 45.99 | 45.56 | 0.18 |
|  | Elevation | -20.7 – 259.0 | 71.5 | 83.2 |
|  | Temperature | 4.93 – 10.57 | 8.01 | 1.15 |
|  | Precipitation | 0.00 – 9.00 | 1.97 | 2.07 |
| B) Change in laying date | | | | |
| SY dataset | ∆Temperature | -2.31 – 3.04 | 0.50 | 1.35 |
|  | ∆Density | -0.50 – 0.50 | 0.00 | 0.20 |
| ASY dataset | ∆Temperature | -3.13 – 4.04 | 0.32 | 1.62 |
|  | ∆Density | -0.70 – 0.40 | 0.00 | 0.16 |
| TOTAL dataset | ∆Temperature | -3.13 – 4.04 | 0.42 | 1.56 |
|  | ∆Density | -0.70 – 0.50 | 0.00 | 0.17 |
| C) Random regression model | Latitude | 45.26 – 45.99 | 45.56 | 0.19 |
|  | Temperature | 4.93 – 10.57 | 8.03 | 1.15 |
|  | Density | 0.10 – 1.00 | 0.75 | 0.22 |

**Table A4** –Linear mixed effects model used to assess if slopes of within-individual (β_W_) and between-individual environmental components (β_B_) are similar or not (following van de Pol and Wright [1], equation 3; see also table 3). The random effect structure was identical to model 5 in the random regression analyses.

| **Estimates of fixed effects** | **Estimate** | **S.E.** | **t-value** | ***P*-value** |
| --- | --- | --- | --- | --- |
| Intercept (β_0_) | 138.605 | 0.752 | 184.41 | <0.001 |
| Age | 7.274 | 0.646 | 11.25 | <0.001 |
| Latitude | 0.638 | 0.303 | 2.11 | 0.048 |
| Temperature_within_ (β_W_) | -1.408 | 0.468 | 3.01 | 0.004 |
| Temperature_between_ - Temperature_within_ (β_B_- β_W_) | 0.414 | 0.472 | 0.88 | 0.38 |
| Density_within_ (β_W_) | -0.347 | 0.421 | 0.82 | 0.41 |
| Density_between_ - Density_within_ (β_B_- β_W_) | -1.039 | 0.500 | 2.80 | 0.039 |

**Reference**

van de Pol M. and J. Wright. 2009. A simple method for distinguishing within- versus between-subject effects using mixed models. Animal Behaviour 77:753–758.

**Appendix S2:** *Additional individual plasticity analyses*

Results from the random regression model and the environmental determinants analyses suggest that habitat with lower density could constraint laying date plasticity in response to spring temperature (see Results section in the main text). To further explore this hypothesis, we conducted additional individual plasticity analyses using our datasets subdivided into high and low breeder densities. These additional analyses were conducted for both plasticity analyses (change in laying date and random regression model) and subsets were created based on the median of mean individual values of observed densities (table S5, table S7) or for the quarter of lower/higher mean individual values of observed densities (table S6, change in laying date analysis only).

**Table B1** – Analyses of change in laying date between two consecutive years by female tree swallows in relationship to change in spring temperature and breeder density for a subset of A) low density of breeders (158 females) and B) high density of breeders (173 females). Subsets were created based on the median of mean individual values of observed densities (18 females with the median were excluded).

| **Model** | **Variable** | **Estimate** | **S.E.** | **t-value** | ***P*-value** |
| --- | --- | --- | --- | --- | --- |
| 1. Low density of breeders | Intercept | -2.037 | 0.694 | 2.94 | 0.004 |
|  | Age | -6.566 | 1.545 | 4.25 | <0.001 |
|  | ∆Temperature | -1.738 | 0.656 | 2.63 | 0.009 |
| 1. High density of breeders | Intercept | -2.697 | 0.608 | 4.43 | <0.001 |
|  | Age | -6.673 | 1.540 | 4.33 | <0.001 |
|  | ∆Temperature | -2.644 | 0.539 | 4.91 | <0.001 |

**Table B2** – Analyses of change in laying date between two consecutive years by female tree swallows in relationship to change in spring temperature and breeder density for a subset of A) low density of breeders (87 females) and B) high density of breeders (80 females). Subsets were divided based on the quartiles of higher/lower of mean individual values of observed densities.

| **Model** | **Variable** | **Estimate** | **S.E.** | **t-value** | ***P*-value** |
| --- | --- | --- | --- | --- | --- |
| 1. Low density of breeders | Intercept | -3.263 | 0.973 | 3.35 | 0.001 |
|  | Age | -5.292 | 2.074 | 2.55 | 0.013 |
|  | ∆Temperature | -2.010 | 0.976 | 2.06 | 0.043 |
| 1. High density of breeders | Intercept | -1.781 | 0.466 | 3.83 | <0.001 |
|  | Age | -3.440 | 1.195 | 2.88 | 0.005 |
|  | ∆Temperature | -3.665 | 0.461 | 7.96 | <0.001 |

**Table B3** – Random regression analyses of the effect within-individual (β_W_) and between-individual (β_B_) components of spring temperature on female tree swallow laying dates for a subset of A) low density of breeders (434 observations on 176 females) and B) high density of breeders (456 observations on 175 females). Subsets are divided based on the median of mean values of observed densities (19 females with the median were excluded). Estimates of fixed effects and variance components of random effects (in bold) are presented.

| **A) Low density of breeders** | | |  | | |  | |  |  | |  | |
| --- | --- | --- | --- | --- | --- | --- | --- | --- | --- | --- | --- | --- |
| **Models** | | | Log-L | | | Test | | d.f. | LRT | | *P*-value | |
| 1**. Year + Farm + Female** | | | -1359.6 | | |  | | 9 |  | |  | |
| 2. Year + Farm + Female X Temperature_within_ | | | -1359.9 | | | 1 vs. 2 | | 11 | 0.03 | | 0.99 | |
| **Estimates of fixed effects** | **Estimate** | **S.E.** | | **t-value** | ***P*-value** | | **Random effects** | | | **Var** | |  |
| Intercept (β_0_) | 139.500 | 0.789 | | 176.79 | <0.001 | | Female (intercept) | | | 7.765 | |  |
| Age | 7.301 | 0.892 | | 8.18 | <0.001 | | Year (intercept) | | | 3.404 | |  |
| Latitude | 0.835 | 0.522 | | 1.60 | 0.12 | | Farm (intercept) | | | 3.923 | |  |
| Temperature_within_ (β_W_) | -1.548 | 0.605 | | 2.56 | 0.016 | | Residual | | | 21.982 | |  |
| Temperature_between_ (β_B_) | -0.785 | 0.658 | | 1.19 | 0.24 | |  | | |  | |  |
| **B) High density of breeders** | | |  | | |  | |  |  | |  | |
| **Models** | | | Log-L | | | Test | | d.f. | LRT | | *P*-value | |
| 1. **Year + Farm + Female** | | | -1410.2 | | |  | | 9 |  | |  | |
| 2. Year + Farm + Female X Temperature_within_ | | | -1410.0 | | | 1 vs. 2 | | 11 | 0.30 | | 0.86 | |
| **Estimates of fixed effects** | **Estimate** | **S.E.** | | **t-value** | ***P*-value** | | **Random effects** | | | **Var** | |  |
| Intercept (β_0_) | 137.866 | 0.739 | | 186.58 | <0.001 | | Female (intercept) | | | 6.527 | |  |
| Age | 7.571 | 1.060 | | 7.14 | <0.001 | | Year (intercept) | | | 3.207 | |  |
| Latitude | 0.415 | 0.409 | | 1.02 | 0.33 | | Farm (intercept) | | | 1.439 | |  |
| Temperature_within_ (β_W_) | -1.904 | 0.572 | | 3.33 | 0.003 | | Residual | | | 21.726 | |  |
| Temperature_between_ (β_B_) | -1.566 | 0.644 | | 2.43 | 0.019 | |  | | |  | |  |
